# VHS, US3 and UL13 viral tegument proteins are required for Herpes Simplex Virus-Induced modification of protein kinase R

**DOI:** 10.1038/s41598-020-62619-2

**Published:** 2020-03-27

**Authors:** Rosamaria Pennisi, Maria Musarra-Pizzo, Zhixiang Lei, Grace Guoying Zhou, Maria Teresa Sciortino

**Affiliations:** 1 Department of Chemical Biological Pharmaceutical and Environmental Sciences, University of Messina, Viale F. Stagno d’Alcontres, 31, Messina, 98166 Italy; 2 Shenzhen International Institute for Biomedical Research, 140 Jinye Ave. Building A10, Dapeng New District, Shenzhen, Guangdong 518116 China; 3 School of Basic Medical Sciences, Guangzhou Medical University, Guangzhou, Guangdong 511436 China

**Keywords:** Herpes virus, Virus-host interactions

## Abstract

To replicate, spread and persist in the host environment, viruses have evolved several immunological escape mechanisms via the action of specific viral proteins. The model “host shut off” adopted by virion host shut off (VHS) protein of Herpes simplex type 1 (HSV-1) represents an immune evasion mechanism which affects the best-characterized component of the innate immunological response, protein kinase R (PKR). However, up to now, the real mechanism employed by VHS to control PKR is still unknown. In this paper, we implement and extend our previous findings reporting that wild-type HSV-1 is able to control PKR, whereas a VHS mutant virus (R2621) clearly induces an accumulation of phosphorylated PKR in several cell types in a VHS-RNase activity-dependent manner. Furthermore, we demonstrate for the first time a new PKR-regulatory mechanism based on the involvement of Us3 and UL13 tegument viral proteins. The combined approach of transfection and infection assay was useful to discover the new role of both viral proteins in the immunological escape and demonstrate that Us3 and UL13 control the accumulation of the phosphorylated form (ph-PKR). Lastly, since protein kinases are tightly regulated by phosphorylation events and, at the same time, phosphorylate other proteins by inducing post-translational modifications, the interplay between Us3 and VHS during HSV-1 infection has been investigated. Interestingly, we found that VHS protein accumulates at higher molecular weight following Us3 transfection, suggesting an Us3-mediated phosphorylation of VHS. These findings reveal a new intriguing interplay between viral proteins during HSV-1 infection involved in the regulation of the PKR-mediated immune response.

## Introduction

The main purpose of all viruses is to guarantee their survival in the cellular environment by overcoming the innate immune response. Among antiviral mechanisms, the protein kinase R (PKR) is the best- characterized Interferon Stimulated Genes (ISGs) protein, composed by two dsRNA binding domains (dsRBDs) in the N-terminal domain and kinase catalytic site in the C-terminal region^1,2^. The expression of PKR is transcriptionally induced by interferon, (IFN) but the kinase activity is promoted by binding to double-stranded RNA (dsRNA) originated by RNA viruses or by dsRNA replicative forms that represent obligatory intermediates for the synthesis of new genomic RNA copies^3^. Instead, the DNA viruses produce overlapping mRNA transcripts that can mimic dsRNAs, which are responsible for PKR activation in infected cells^4^. It was theorized that PKR exists in a closed conformation in which the dsRBD overlays the kinase catalytic site and auto-inhibits binding with substrates^5^. The dsRNA interaction pushes dsRBM2 out of the kinase domain and exposes the catalytic site for autophosphorylation at Thr446 residue. The autophosphorylation occurs with an intermolecular mechanism, which involves the dimerization of two monomers of PKR, structural rearrangement, the shift from inactive to activate forms and the recognition and the phosphorylation of the alpha subunit of eIF2 translation initiation factor^6^. All viruses depend on cellular protein synthesis machinery to translate viral messenger RNAs and produce new viral progeny^7^. The shutdown of overall protein synthesis caused by PKR-mediated eIF2α phosphorylation is virally counteracted by several immune escape strategies^8–26^. Non-coding RNAs^8^, dsRNA-binding proteins^10,14^, and post-transcriptional regulators^16,17^ produced by viruses belonging to several families inhibit the PKR activation or its downstream effect. Many viral proteins involved in this process have structural functions other than their equivalents in other viruses, such as Picornavirus proteases, which also have a gene expression regulatory function^19^. The regulatory role of the tegument proteins as well as their structural function has interested several researchers on their potential involvement in immune response evasion. Early studies have shown that during viral replication, HSV-1 accumulates viral proteins and gradually reduces the production of host proteins, progressively^27^. This phenomenon, known as “host shutoff”, was achieved by virion host shutoff protein (VHS) protein of HSV-1; its structural role is strictly associated with an endoribonuclease activity responsible for the degradation of host mRNAs and the turnover of viral mRNAs^28–31^. The inability to discriminate cellular mRNAs and viral mRNAs makes VHS an uncontrolled threat for viral survival^32^. Therefore, the trimeric complex with VP22 (encoded by the UL49 gene) and VP16 (encoded by the UL48 gene) and/or the association with UL47 HSV-1 proteins modulates the intracellular levels of VHS and define the viral regulatory mechanism responsible for gene cascade activation and preserves viral mRNAs from degradative activity^33–36^. Then, VHS affects the host mRNAs integrity in a selective dynamic manner by degrading mRNAs containing AU-rich elements in the 3′UTR and via shuttling between nuclear and cytoplasmic compartments^28–30^. The model of “host shutoff” switches the gene expression machinery for the viral gene cascade and represents an immune evasion mechanism which counteracts the activation of PKR^37–39^. Although VHS’s role has been outstandingly described in destabilizing host mRNAs^40^, it is not perfectly clear whether VHS works alone, or if it activates cellular enzymes with an RNAse function, or if it recruits other viral proteins directly or indirectly to improve its activity^31^.

## The involvement of Us3 and UL13 tegument viral proteins in PKR regulation

The present study stemmed from the observation that HSV-1 encodes tegument proteins with additional kinase enzymatic activities useful in the innate immune escape mechanism^41–61^. Therefore, we hypothesized the involvement of Us3 and UL13 serine/threonine protein kinases in the regulation of PKR. The Us3 and UL13 gene products are tegument viral proteins with kinase activities involved in the viral transport and maturation^41^. Phosphorylation events that are mainly catalyzed by viral protein kinases represent post-translational modifications useful for the regulation of viral proteins and promote efficient viral replication^42^. Us3 is one of the best-characterized HSV-1 proteins involved in several processes, including egress of virus particles from the nucleus and the modulation of the actin cytoskeleton to promote virion spread and inhibition of apoptosis^43,44^. The main functions associated with HSV-1 Us3 protein kinase are correlated with the phosphorylation of cellular and viral substrates^45–49^. The catalytic activity of Us3 is tightly regulated by autophosporylation events on residue Ser-147 and by the phosphorylations mediated by UL13^50,51^. The UL13 kinase activity regulates the expression of viral proteins^52–60^, promotes viral replication as well as the assembly and release of virions^41^. The failure in the expression of UL13 kinase provokes deeper profound effects on viral egress in several cell lines than the loss of Us3 kinase, suggesting an unequal contribution from each of the kinases to viral egress. In the context of immune response, the deletion of the *ul13* gene enhances susceptibility to type I IFN, proposing an immunological escape mechanism correlated to kinase activity of UL13^61^. In our opinion, the combined activity of the HSV-1 protein kinases play an important role in viral replication by overcoming the host immune barriers. Therefore, in the present study, we used a combined approach of viral plasmid transfection and mutant virus infection to investigate the role of VHS, Us3 and UL13 in the regulation of PKR. We demonstrate for the first time that Us3 and UL13 proteins play an important role in inhibiting the accumulation of the phosphorylated form of PKR. Furthermore, we have been able to demonstrate a new potential regulatory mechanism, which involves the interplay between VHS and Us3 on the regulation of PKR-mediated response.

## Results

### The viral protein VHS controls the PKR phosphorylation levels in different cell lines

**Figure 1:**
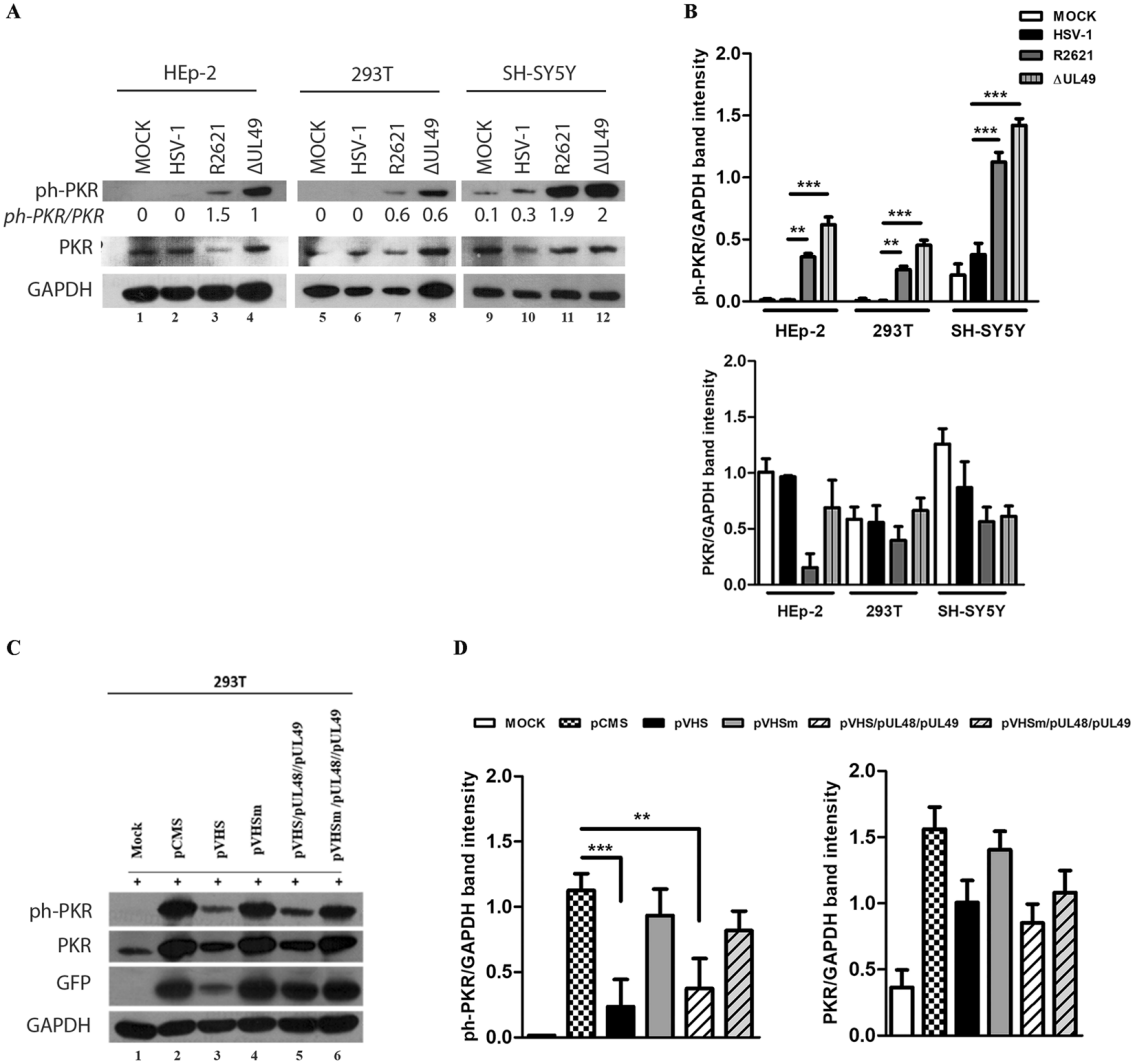
Evaluation of total PKR and ph-PKR in different cell lines infected with HSV-1, R2621 and ΔUL49 mutant viruses. (**a**) HEp-2, 293T and SH-SY5Y were infected with HSV-1, vhs-deleted (R2621) and ΔUL49 mutant viruses at MOI 10 and harvested 24 h p.i. The samples were subjected to western blot to analyse both total PKR and ph-PKR. GAPDH was used as a loading control. (**b**) Quantification of band intensity was determined with T.I.N.A program, the ph-PKR and PKR values were normalized to GAPDH protein levels and graphically represented using GraphPad Prism 6 software (GraphPad Software, San Diego, CA, USA). Statistical significance was tested by one-way analysis of variance (ANOVA) (*p < 0.05, **p < 0.01, and ***p < 0.001). (**c**) Transient transfection of VHS and mutant VHS plasmids in 293T cells. The cells were transfected with 1 μg of plasmid DNA as described in Methods. Then, 48 h post transfection, the cells were left untreated or treated with poly I:C (0,01 μg/μl) for an additional 24 h. Western blot analysis was carried out to analyse ph-PKR, PKR, GFP and GAPDH expression following transfection with pCMS-EGFP (symbolised in the figure as pCMS), pVHS, pVHSm, pVHS/pUL48/pUL49 and pVHSm/pUL48/pUL49. (**d**) The quantification of band intensity of ph-PKR and PKR were normalized to GAPDH protein levels and graphically represented by GraphPad Prism 6 software (GraphPad Software, San Diego, CA, USA). Statistical significance was tested by one-way analysis of variance (ANOVA) (**p < 0.01, and ***p < 0.001).

As a component of antiviral responses, PKR acts by inhibiting protein synthesis initiation and by activating the transcription of genes involved in the inflammatory response. For this reason, the control of PKR represents a fundamental strategy to avoid the innate immune response and promote viral survival. Accumulating evidence suggest that the VHS protein controls PKR activation with a not-yet-fully understood mechanism. Since it has been previously reported that VHS abrogates the accumulation of ph- PKR in HT1080 cells^38^, the first objective of our study was to verify the impact of VHS on ph-PKR accumulation in different cell lines. In addition, we assessed whether the enzymatic nature of VHS could regulate PKR activation by using a genetic approach, which alters the nuclease activity of VHS. Therefore, HEp-2, 293T and SH-SY5Y cell lines were mock infected and infected at MOI 10 with HSV-1, or mutant viruses deleted for *vhs* gene (R2621) or for the *ul49* gene (ΔUL49) which exhibits an abrogation of VHS’s RNase activity^35^. The cells were collected 24 h post infection (p.i.) and total cellular extracts were subjected to western blot analysis to evaluate the accumulation of total and phosphorylated form of PKR by using a primary antibody directed against phosphorylated residue Thr-446 in the activation loop where the autophosphorylation site is mapped. As shown in Fig. 1a, the HEp-2, 293T and SH-SY5Y cell lines infected with HSV-1 wild-type lack in the accumulation of phosphorylated form of PKR if compared to uninfected cells (Fig. 1a lanes 2, 6, 10). On the contrary, high phosphorylation levels of PKR were observed following both R2621 (Fig. 1a lanes 3, 7, 11) and ΔUL49 (Fig. 1a lanes 4, 8, 12) viruses. The obtained results were complemented by quantitative analysis of the band-intensity of ph-PKR on GAPDH levels and PKR on GAPDH levels (Fig. 1b). The accumulation of ph-PKR, following infection with both mutant viruses, coincides with the simultaneous reduction of total form of PKR. In addition, the ratio between ph-PKR/PKR, as reported in Fig. 1a, shows the switch of the total form of PKR to the ph-PKR, predominantly in HEp- 2 and SH-SY5Y cells infected with R2621 and ΔUL49 viruses. In cells infected with HSV-1, the expression levels of both ph-PKR and total PKR remains unchanged compared to the uninfected cells. These findings demonstrate that HSV-1 is capable of controlling the accumulation of ph-PKR in different cell lines with a mechanism that involves mainly the VHS RNase activity. Indeed, the deletion of vhs gene in R2621 and the natural loss of VHS RNase enzymatic function belonging naturally in ΔUL49 implies the failure of the control of the PKR-mediated response during HSV-1 replication. To support this finding, transient transfection experiments were performed by using plasmids expressing the wild type VHS and mutant VHS, designed as pVHSm, alone and/or in triple transfection with pUL48 and pUL49. The pVHSm is characterized by substitutions of three amino acid residues, E192A, D194A, and D195A, which are essential for the nuclease activity^36^. Therefore, the pVHSm encodes a catalytically inactive form of the VHS protein. In addition, the pUL48 and pUL49 plasmids, able to overexpress UL48 and UL49 HSV-1 proteins, were chosen because, according to literature data, they are able to control the expression of VHS protein^62^. The 293T cells were transfected with 1 μg of DNA plasmids for 48 h and treated with poly I:C for further 24 hours. It has been demonstrated that poly I:C, a synthetic analogue of double-stranded RNA (dsRNA), behaves like an inductor of PKR by decreasing the activation of pre-existing PKR^63^. The expression of the total and phosphorylated forms of PKR was examined and compared to cells transfected with pCMS-EGFP plasmid, used as a control vector. Nejepinska and co-authors has been previously reported that the transfection of plasmid DNA allows the transcription of spurious dsRNAs, which can activate PKR^64^. Therefore, the simultaneous transfection of pCMS-EGFP as a control vector and poly I:C represents a double stimulus to PKR response which allows us to investigate VHS’s role. In addition, the pCMS-EGFP protein accumulation was used to monitor the transfection efficiency of the 293T cells. Figure 1c shows the reduction of ph-PKR accumulation in poly I:C-treated cells transfected with pVHS plasmid alone and in triple transfection with pUL48 and pUL49 plasmids, compared to cells transfected with pCMS-EGFP alone (Fig. 1c lanes 3 and 5 vs lane 2). On the contrary, an accumulation of ph-PKR was detected in pVHSm transfection, alone and in triple transfection with pUL48 and pUL49 plasmids, if compared to pVHS (Fig. 1c lanes 4 and 6 vs 3 and 5). The quantitative analysis of the band intensity related to ph-PKR accumulation is graphically reported in Fig. 1d. Overall, the transfection experiments showed the reduction of the total form of PKR (Fig. 1c) when the VHS protein is active compared to mutated VHS protein, indicating a crucial role of the RNase activity of VHS on PKR activation.

### The accumulation of viral mRNA is responsible for PKR activation

**Figure 2:**
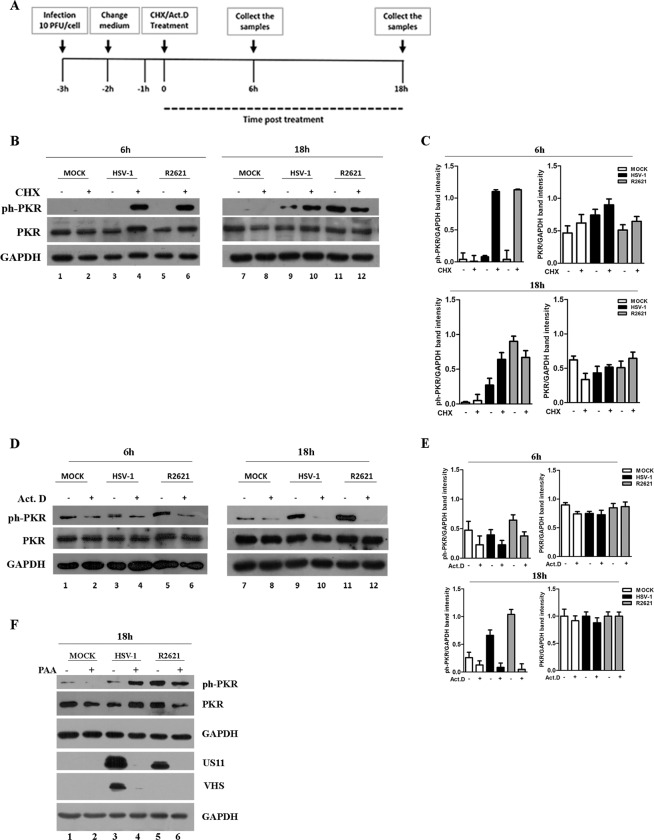
Detection of phosphorylated PKR and total PKR during viral replication in cells treated with protein, mRNA and viral DNA synthesis inhibitors. (**a**) Graphical representation of the experiment. (**b–d**) HEp-2 cells were mock infected and infected with HSV-1 (F) and R2621 viruses. Three hours p.i., the cells were left untreated or treated with Cycloheximide (CHX) (50 μg/ml) or actinomycin D (Act.D) (10 μg/ml), separately, and collected at 6 h and 18 h of treatment. Western blot analysis was performed to evaluate ph-PKR, PKR and GAPDH expression. The grouping blots are cropped from two different gels, as displayed in the figure with the white space. (**c**–**e**) Band density was determined with the T.I.N.A. program, expressed as fold change over the appropriate housekeeping genes and graphically represented by GraphPad Prism 6 software. (**f**) HEp-2 cells were mock infected or infected with HSV-1 and R2621 viruses at MOI 10, and collected at 24 h p.i. PAA was used at 300 μg/mL into the inoculum, and maintained in the medium.

The activation mechanism of PKR is triggered upon binding to double-stranded RNA (dsRNA) which induces the dimerization, the autophosphorylation and a structural rearrangement of the protein responsible for the exposure of the catalytic site. Previously, an activation model of PKR based on the optimal concentration of RNAs able to activate or inhibit it has been proposed^3,5^. This mechanism represents a powerful strategy by which several viruses counteract PKR functions. In the context of HSV-1 infection, the above function could be exerted by the ribonuclease activity of VHS. To verify the involvement of the overloaded mRNAs in PKR activation, two pharmacological inhibitors of protein synthesis were used. Figure 2a shows the graphical representation of the experiments. HEp-2 cells were mock infected and infected at MOI 10 with HSV-1 (F) wild-type or R2621 viruses. Three hours later, uninfected and infected cells were separately treated with cycloheximide (CHX) (50 μg/ml) or with actinomycin D (Act.D) (10 μg/ml). Then, samples were harvested at 6 h and 18 h of treatment. The CHX treatment was used to inhibit protein synthesis, which provokes an overload of mRNAs^65^. The Act.D was used to block transcription that causes the decline of mRNAs accumulation^66^. The key features of the results presented in Fig. 2 were as follows: (i) high accumulation of ph-PKR was detected in both HSV-1- and R2621- infected cells treated with CHX if compared to the mock cells at 6 h and 18 h (Fig. 2b lanes 4, 6, 10, 12). The results suggested that in the examined cellular model, the overload of viral mRNAs triggers the ph-PKR accumulation. (ii) in CHX-untreated cells, the accumulation of ph-PKR was absent at 6 h in cells infected with R2621 as well as with HSV-1 (Fig. 2b lanes 3 and 5). At 18 h, when high synthesis of VHS protein occurs, in CHX-untreated infected cells, the ph-PKR accumulation decreases in cells infected with HSV-1 rather than R2621 (Fig. 2b lane 11 vs 9), confirming the role of VHS in the control of PKR phosphorylation. (iii) The Act.D treatment provoked the reduction of ph-PKR in the treated-infected cells compared to untreated-infected cells in both viruses (Fig. 2b lanes 4, 6, 10 and 12), pointing out the importance of mRNAs accumulation in the activation of PKR. The quantitative analysis of the band intensity related to ph-PKR and total PKR expression during HSV-1 replication and following CHX and Act. D treatment is graphically reported in Fig. 2c,e. Taken together from these data, we can conclude that the accumulation of viral RNAs and then the *de novo* synthesis of viral proteins is required to control ph-PKR. To discriminate if the transcription of early or late genes was responsible for the ph-PKR control, the expression of γ_2_ genes was inhibited by treating cells with DNA polymerase inhibitor phosphonoacetic acid (PAA, 300 μg/ml). HEp-2 cells were infected or mock-infected with both HSV-1 and R2621 at MOI 10, in presence of PAA and samples were collected at 18 h p.i. The results, reported in Fig. 2 panel f, showed that ph-PKR highly accumulates in presence of PAA in HSV-1 cells compared to untreated infected cells (Fig. 2f lane 4 vs 3) indicating that the accumulation of γ_2_ genes during HSV-1 replication is responsible for the ph-PKR control. Indeed, the expression of VHS was blocked by PAA treatment (Fig. 2 f lane 4 vs 3). Overall, these data indicate that at 24 h the lack in VHS protein expression, due to PAA treatment, overlaps the HSV-1 phenotype with the R2621 phenotype with respect to the accumulation of ph-PKR (Fig. 2f lane 4 vs 5).

### Involvement of viral proteins Us3 and UL13 in the accumulation of ph-PKR

**Figure 3:**
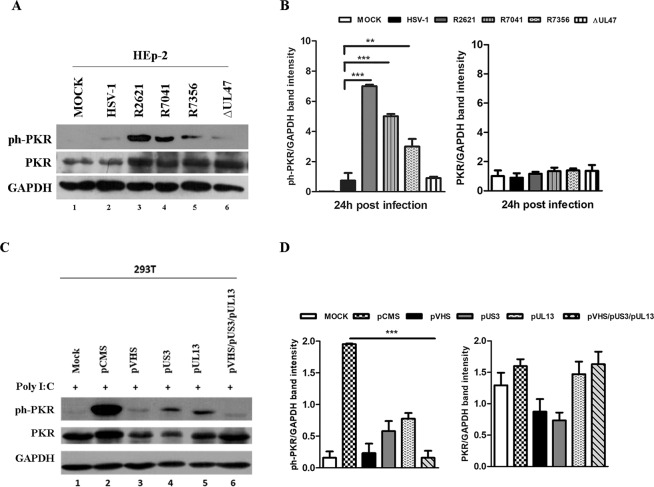
Detection of total PKR and ph-PKR in infection or transfection experiments. (**a**) HEp-2 cells were mock infected or infected with HSV-1 wild-type virus, R2621 (ΔVHS), R7041 (ΔUs3), R7356 (ΔUL13) mutant viruses at MOI 10 and harvested 24 h p.i. The ∆UL47 virus was used as a viral control. Western blot analysis was performed to detect ph-PKR (Thr-446), PKR and GAPDH expression. (**b**–**d**) Band density was determined with the T.I.N.A. program, expressed as fold change over the appropriate housekeeping genes and graphically represented by GraphPad Prism 6. Statistical significance was tested by one-way analysis of variance (ANOVA) (**p < 0.01, and ***p < 0.001). (**c**) 293T cells were transfected with 1 μg of plasmid DNA according to manufacturer’s instructions. 48 h post transfection the cells were treated with poly I:C (0,01 μg/μl) for an additional 24 h. Western blot analysis was carried out to analyse ph-PKR, PKR and GAPDH expression.

To understand VHS-mediated regulation of PKR, an investigation of physical interactions among these two proteins was required. Nevertheless, the lack of evidence that VHS protein interacts physically with PKR (data obtained in our laboratory but not shown) raised the question whether additional mechanisms and additional viral proteins were involved in the PKR response during HSV replication. In this context, in addition to conventional structural roles, the HSV-1 tegument proteins play multiple roles during the viral life cycle. Indeed, in infected cells, they are modulators of cellular and viral functions and perform different enzymatic activities to improve viral replication. How these are coordinated during infection is largely unknown. Therefore, based on the above considerations, the regulatory role of two HSV-1 tegument proteins, Us3 and UL13, on the PKR was investigated. Thus, deleted viruses for *us3* and *ul13* genes and plasmids expressing Us3 and UL13 proteins were employed to verify their involvement in the PKR accumulation during HSV-1 replication. First, HEp-2 cells were mock infected and infected with HSV-1 wild-type virus, R2621, R7041 and R7356 mutant viruses, separately, at MOI 10 and harvested at 24 h p.i. R7041 and R7356 differs from the wild-type HSV-1 solely with respect to the deletion in the Us3 and UL13 open reading frame, respectively^56,67^. The ∆UL47 virus was used as a control in which the failure of the *ul47* viral gene expression was not involved in the proposed mechanism. The results, shown in Fig. 3a, demonstrate for the first time that cells infected with the viruses deleted for *us3* and *ul13* genes highly accumulate ph-PKR. The ph- PKR accumulation was greater in R2621, R7041 and R7356 compared to HSV-1 (Fig. 3a, lanes 3, 4 and 5 vs 2). The quantitative analysis of the band intensity related to ph-PKR were graphically reported in Fig. 3b. To confirm these data, transient transfection experiments were carried out in 293T cells. The cells were transfected with plasmids encoding VHS, UL13 and Us3 viral proteins and 48 h after transfection, the cells were treated with poly I:C (0,01 μg/μl) for an additional 24 h. Then, cells were collected and subjected to western blot analysis. The expression of both total and phosphorylated forms of PKR were analysed and compared to cells transfected with pCMS-EGFP plasmid used as a control vector. According to the previous results, VHS expression, by transfection, reduced significantly the accumulation of the phosphorylated form of PKR if compared to the control vector (Fig. 3c lane 3). Interestingly, low accumulation levels of ph-PKR were detected after single transfection with pUS3 and pUL13 (Fig. 3c lanes 4 and 5). However, the triple transient transfection with pVHS, pUS3 and pUL13 shows a strongly reduction of accumulation of ph-PKR (Fig. 3c lane 6). The quantitative analysis of the band intensity related to ph-PKR is graphically reported in Fig. 3d. It was also noted that the transfection with plasmids encoding for VHS and US3 significantly affects the expression of total form of PKR compared to the basal levels in control cells (Fig. 3c lanes 3 and 4 vs 1). These findings, associated to the results obtained in the viral infection experiments (Fig. 3a), demonstrated for the first time the involvement of additional HSV-1 structural proteins in the regulation of both total and ph-PKR accumulation.

### The regulation of PKR matches with the temporal accumulation of Us3 and VHS at the late stage of viral replication

**Figure 4:**
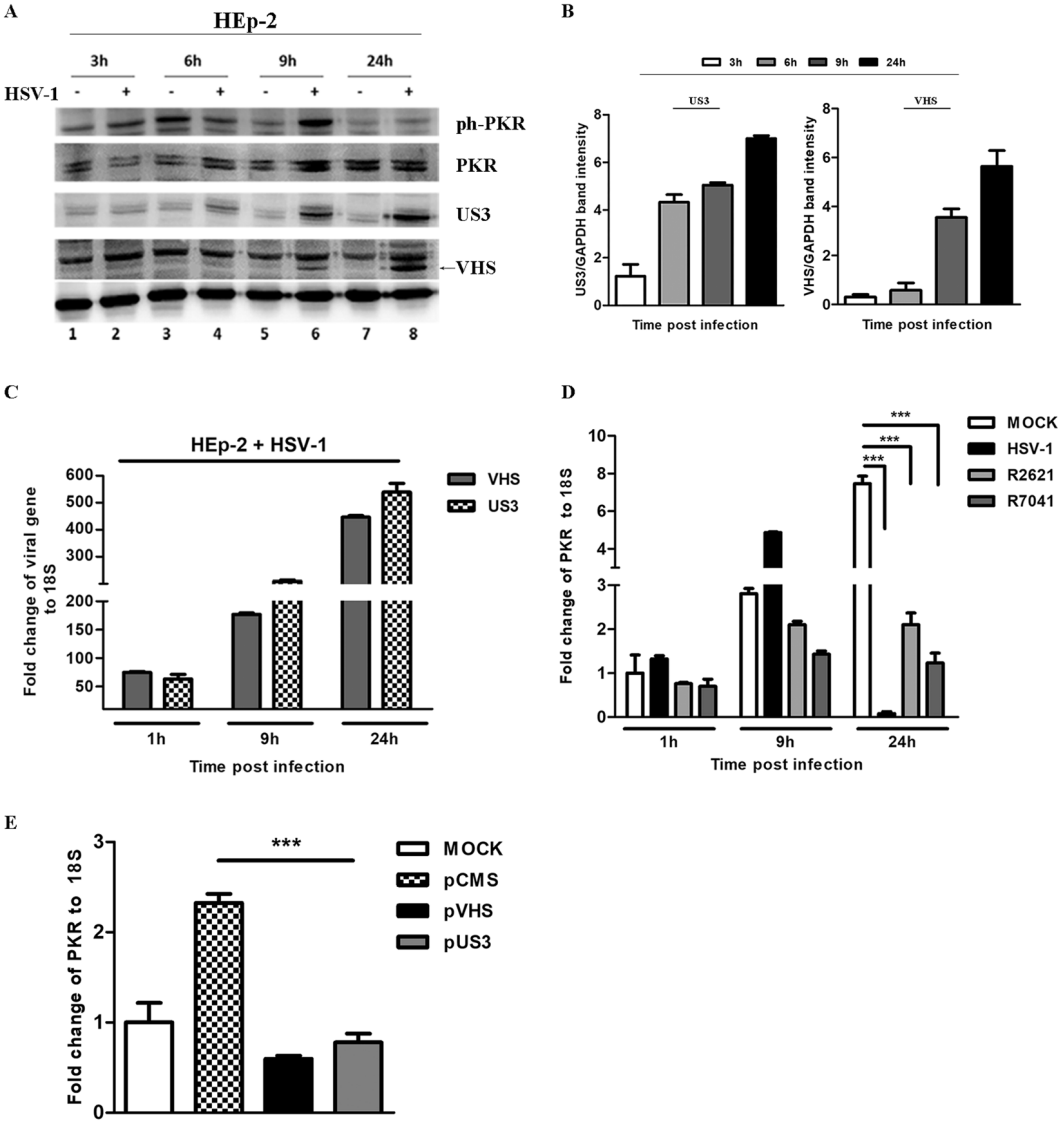
The regulation of PKR matches with the temporal accumulation of Us3 and VHS in HEp-2 cells infected with HSV-1. (**a**) HEp-2 cells were mock infected and infected with HSV-1 at MOI 10 and collected at 3 h, 6 h, 9 h, 24 h p.i. The protein extracts were separated by polyacrylamide gel electrophoresis and the ph-PKR, PKR, Us3 and VHS expression levels were detected. The GAPDH was used as a housekeeping control. Protein bands were captured using a ChemiDoc Touch Imaging System (Bio-Rad). (**b**) Band density was determined with the T.I.N.A. program, expressed as fold change over the appropriate housekeeping genes and graphically represented by GraphPad Prism 6 software (GraphPad Software, San Diego, CA, USA). (**c**) Quantitative Real-time PCR was performed to evaluate the *vhs* and *Us3* transcripts in HEp-2 cells infected with HSV-1 at MOI 10 at 1 h, 9 h and 24 h p.i. (**d**) Quantitative Real-Time PCR was performed to detect the levels of PKR transcripts during viral replication. HEp-2 cells were mock infected or infected with HSV-1, R2621 and R7041 viruses at MOI 10 and collected at 1 h, 9 h, and 24 h p.i. (**e**) 293T cells were transfected with pVHS and pUS3 plasmids and pCMS-EGFP was used as a control vector. Total RNA extractions were carried out as described in Methods. Real-Time PCR was used to detect the PKR transcripts in presence or absence of specific structural HSV-1 proteins. The levels of PKR and 18S rRNA, used as an endogenous control, were evaluated by using the comparative CT Method (ΔΔCT Method) as described in Materials and Methods. Statistical analyses were performed with one-way analysis of variance (ANOVA) in triplicate and ***P < 0.001 indicates significant changes.

The findings reported above have aroused the curiosity to verify whether the reciprocal regulation between the viral proteins could be involved in the control of PKR, focusing our attention on VHS and Us3 proteins. Therefore, a time course has been performed to analyze the concomitant expression of Us3 and VHS proteins with the accumulation of the ph-PKR. Thus, HEp-2 cells were mock infected or infected with HSV-1 at MOI 10 and collected at 3 h, 6 h, 9 h, and 24 h p.i. The results, shown in Fig. 4, demonstrate that: (i) the Us3 protein accumulates during the late stage of viral replicative cycle, beginning at 6 h, (Fig. 4a lanes 4, 6 and 8) and VHS protein beginning at 9 h (Fig. 4a lanes 6 and 8). (ii) the decrease of ph-PKR at 24 h p.i. corresponds to VHS and Us3 accumulation during viral infection (Fig. 4a lane 8). The quantitative analysis of the band intensity related to VHS and Us3 expression is graphically reported in Fig. 4b. Quantitative Real-Time PCR analysis was also performed to analyze the accumulation of Us3 and VHS transcripts. As depicted in Fig. 4c, a high increase of vhs and Us3 transcripts was observed at 9 h and 24 h (Fig. 4c). Having demonstrated the concomitant reduction of ph-PKR accumulation with the high expression of Us3 and VHS proteins, the next step was determining whether the viral control on PKR could be limited to protein phosphorylation events or whether it also affects the transcript levels of PKR. Therefore, HEp-2 cells were mock infected and infected with HSV-1, R2621 and R7041 viruses at MOI 10 and collected at 1 h, 9 h and 24 h p.i. Quantitative Real-Time PCR was performed to measure PKR transcript levels. The results, reported in Fig. 4d, showed that during the early stage of viral replication, PKR transcripts accumulate (9 h p.i.) compared to the mock. Additionally, at 24 h p.i., the levels of PKR transcripts drastically decrease in cells infected with HSV-1 but not in cells infected with R2621 and R7041 mutant viruses, with different degrees. Because the expression and activities of both viral and cellular proteins during viral replication are subjected to different types of regulation strategies mediated by onset of viral proteins and by phosphorylation networks, an *in vitro* transfection approach was used to verify the involvement of each tegument protein in the regulation of PKR transcripts. Experiments were carried out in 293T cells using plasmids encoding VHS and Us3 viral proteins. Data shown in Fig. 4e demonstrated that both VHS and Us3 proteins, separately, were able to reduce the levels of PKR transcripts if compared to cells transfected with pCMS-EGFP control vector. This finding demonstrated that the accumulation of VHS and Us3 transcripts (Fig. 4c) and proteins (Fig. 4a), correlates with PKR depletion at the transcriptional and translational level.

### The interplay between viral proteins VHS and Us3 is responsible for regulation of PKR

**Figure 5:**
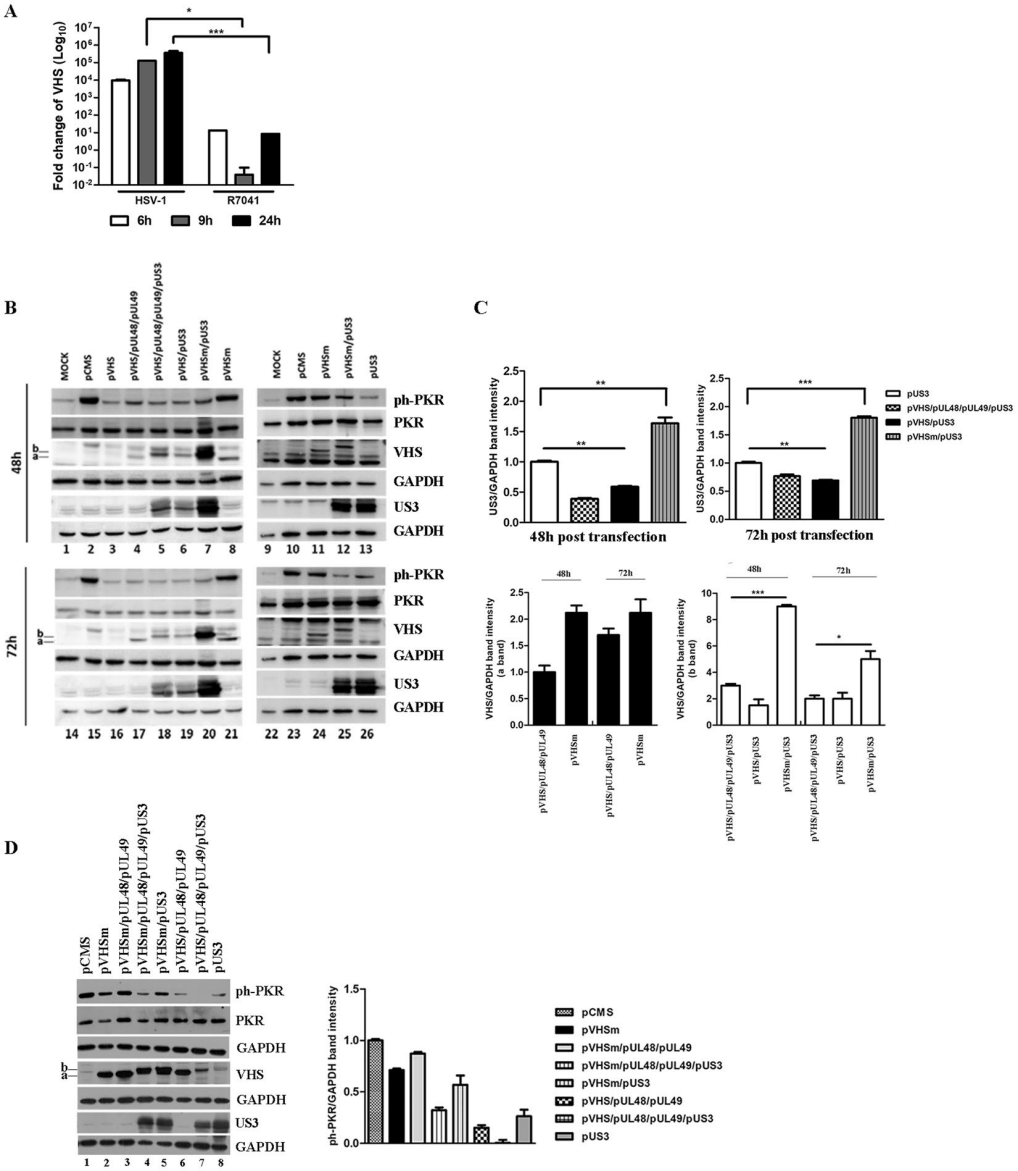
Analysis of VHS and Us3 proteins. (**a**) HEp-2 cells were infected with HSV-1 and R7041 viruses at MOI 10, collected at different time points (6 h, 9 h, and 24 h) and subjected to Quantitative Real-Time PCR was performed to detect VHS transcripts. (**b**) 293T cells were transfected with a combination of plasmids encoding VHS, VHSm, UL48, UL49, and Us3 viral proteins. The pCMS-EGFP plasmid was used as a control vector. The cells were harvested at 48 h and 72 h and the expression of ph-PKR, PKR, VHS and Us3 proteins was evaluated by western blot analysis. The (**a**,**b**) indicate the lower and the higher VHS protein bands, respectively. The grouping blots are cropped from different gels as displayed in figure with white spaces and were captured using a ChemiDoc Touch Imaging System (Bio-Rad). The expression of each protein was simultaneously measured at 48 h and 72 h. (**c**) Band density of Us3 and VHS (a and b bands) was determined with the T.I.N.A. program, expressed as fold change over the appropriate housekeeping genes and graphically represented with GraphPad Prism 6 software. Statistical significance was tested using one-way ANOVA (^***^p < 0.001,*p < 0.05). (**d**) 293T cells were transfected with a combination of plasmids encoding for VHSm, UL48, UL49, and Us3 viral proteins. The pCMS-EGFP plasmid was used as a control vector. The cells were harvested at 48 h and the expression of ph-PKR, PKR, VHS and US3 proteins was evaluated by western blot analysis. The band intensity of ph-PKR and PKR was expressed as fold change over the appropriate housekeeping genes and graphically represented.

A crucial point in the regulatory mechanism that emerged in this work was to understand how the viral proteins regulate each other and how this interaction was involved in the PKR regulation. Therefore, we wanted to monitor at the transcriptional level the accumulation of VHS transcripts in cells infected with Us3-deleted virus compared to those infected with HSV-1. HEp-2 cells were infected with HSV-1 and R7041 at MOI 10 and collected at different time points (6 h, 9 h, and 24 h), and Quantitative Real-Time PCR was performed to analyze the transcript levels of VHS. The results, shown in Fig. 5a, demonstrated that the levels of VHS transcripts were significantly lower in R7041-infected cells at all times considered compared to HSV-1-infected cells. These findings suggest that the lack of Us3 protein negatively affects the accumulation of VHS transcripts. Moreover, in dependence on the kinase enzymatic activity of Us3 and/or the myriad of viral and host substrates phosphorylated directly by Us3, we focused our attention on capability of Us3 to use VHS as substrate. Thus, 293T cells were transfected with a combination of plasmids encoding VHS, UL48, UL49, VHSm and Us3 viral proteins. The UL48 and UL49 plasmids were used to accumulate the VHS protein as described in Taddeo and collaborators^62^. The pCMS-EGFP plasmid was used as a control vector. The cells were harvested at 48 h and 72 h post-transfection and the accumulation of VHS, Us3, PKR and ph-PKR proteins were detected by western blot analysis (Fig. 5b). The results were as follows: (i) at 48 h and 72 h in cells co- transfected with pVHS/pUL48/pUL49, the VHS antibody reacted with a specific band, identified with (a), in presence of pUL48 and pUL49 (Fig. 5b lanes 4 and 17). A similar band was detected in cells transfected with mutant VHS (Fig. 5b lanes 8, 11, 21 and 24); (ii) in cells co-transfected with pVHS/pUL48/pUL49 in presence of pUs3, the VHS antibody reacted with band shifted at high molecular weight, identified with (b) (Fig. 5b lanes 5 and 18). A similar band was detected in cells transfected with pVHSm in presence of pUs3 (Fig. 5b lanes 7, 12, 20 and 25); (iii) interestingly, in cells co-transfected with pVHS/pUs3, for the first time we can visualized an accumulation of VHS protein in absence of UL48 and UL49. In particular, the VHS antibody reacted with band *shifted* at high molecular weight identified with (b) (Fig. 5b lanes 6 and 19). To better evaluate the reciprocal regulation of VHS and Us3, in the same experiment, the accumulation of both proteins was analysed. The results demonstrated that: (i) the VHS protein does not accumulate when cells are transfected with pVHS alone (Fig. 5b lanes 3 and 16); (ii) in cells co-transfected with pVHSm and pUs3, a significant accumulation of higher molecular weight VHS band (b) was recognized (Fig. 5b lanes 7, 12, 20 and 25); (iii) in cells transfected with pVHS/pUs3, an accumulation of Us3 proteins was detected. In particular, a high accumulation of Us3 protein was detected in cells co-transfected with pVHSm and pUs3 if compared the cells co-transfected with pVHS/pUL48/pUL49 or with pVHS alone (Fig. 5b lanes 7, 12, 13, 20, 25 and 26 vs 5, 6, 18 and 19). The Us3 protein accumulates higher in cells transfected with pVHSm compared to pVHS/pUL48/pUL49 or in the double transfection with pVHS. The quantitative analysis of the band intensity related to Us3 and VHS proteins is graphically reported in Fig. 5c. Because UL48 and UL49 damp down VHS activity, allowing translation and accumulation of VHS and their own proteins, we also performed transfection experiments using pVHSm. As shown in Fig. 5d, it was demonstrated that: (i) at 48 h, in cells co-transfected with pVHSm/pUL48/pUL49 in presence of pUs3, the VHS antibody reacted with band shifted at high molecular weight identified with (b) (Fig. 5d, lanes 4–5); (ii) at 48 h, Us3 protein highly accumulates in quadruple transfection with pVHSm if compared to pVHS/pUL48/pUL49/pUs3 (Fig. 5d lanes 4, 5, and 8 vs 7). To the end, it is plausible to hypothesize a new regulatory mechanism on Us3 controlled by VHS RNase activity, as well as a new post-translational phosphorylation modification on VHS protein mediated by Us3. In this context, the accumulation of ph-PKR protein occurs only in cells transfected with pCMS-EGFP or in pVHSm (Fig. 5b lanes 2, 8, 10, 11, 15, 21, 23 and 24; Fig. 5d lanes 1, 2, 3 and 5) as reported also in previous transfection experiments presented in Figs. 1c and 3c.

## Discussion

The regulation of ph-PKR represents a focal point in the innate immune escape mechanism adopted by viruses. Many viruses, including HSV-1, create dsRNAs as replicative intermediates of viral genome replication or from transcription of antisense overlapping genes, this replicative phase serves as sensors with consequent activation of innate immune response. HSV-1 counteracts the PKR-mediated innate immune response by expressing a late gene, Us11, early, which binds and blocks PKR^26^. In addition, it has been demonstrated that HSV-1 encodes the ICP34.5 protein, which recruits the phosphatase alpha and dephosphorylates eIF-2α to preserve viral protein translation and indirectly to block PKR activity downstream of its activation^25^. Furthermore, it was demonstrated that during HSV-1 infection, the MEK protein, normally involved in the cell survival, is engaged in PKR control. Indeed, based on the earlier results performed by Smith and collaborators^37^, the activation of MEK following HSV-1 infection, correlated with suppression of activation of PKR. Moreover, Sciortino and co-authors have demonstrated the involvement of VHS in this regulatory mechanism^38^. These findings, correlated to earlier results by Pasieka and colleagues^39^ showing enhanced levels of phosphorylated eIF2a during VHS-mutant virus infection, highlight that VHS plays a significant role in the blocking of PKR activation.

The key findings reported here complement and extend the studies reported by Sciortino and collaborators. First, we found that the accumulation of ph-PKR occurs in different cell lines with a VHS-dependent mechanism suggesting that HSV-1 has evolved a non-cell specific anti-PKR mechanism during viral replication. A combined infection and transfection approach clearly demonstrated that the lack of VHS RNAse activity, in R2621 and ∆UL49 deleted viruses and with a plasmid encoding a nuclease mutant VHS, affects the negative regulation of ph-PKR mediated by HSV-1 (Fig. 1). Therefore, the VHS endonuclease activity and the subsequent withdrawal of mRNAs induced by VHS were responsible for the ph-PKR accumulation control. Additionally, based on PKR activation model which proposes an optimal concentration of RNAs able to activate or inhibit PKR^3,5^, we have used protein synthesis pharmacological inhibitors to reproduce *in vitro* the PKR activation. Cycloheximide and actinomycin D are respectively able to block the translational elongation mechanisms with consequent accumulation of all form mRNAs and block transcription, which culminates in the shutdown of viral protein synthesis^65,66^. We found that during viral infection, the overload of mRNAs following CHX treatment promotes and accelerates the ph-PKR accumulation (Fig. 2b). The actinomycin D treatment, used to inhibit the transcription, abrogated the ph-PKR accumulation (Fig. 2d). An accumulation of ph-PKR was detected with or without an overload of mRNAs in R2621-infected cells, indicating that VHS can act on mRNA accumulation during viral infection and allows HSV-1 to control PKR. Furthermore, we report that the absence of VHS protein due to PAA treatment makes the HSV-1 phenotypes similar to R2621 in respect to ph-PKR accumulation (Fig. 2f). Alternatively, PAA treatment could interfere with the accumulation of partially complementary transcripts. Indeed, Smiley and co-authors’ in a parallel work demonstrated that partially complementary transcripts from the UL23/UL24 and UL30/U31 regions of the viral genome trigger ph-PKR accumulation^68^. In particular, by developing a plasmid-based system, they produce defined dsRNA species in an uninfected cellular system and provide direct evidence that VHS limits the accumulation of dsRNA, including species arising from complementary viral transcripts. Additionally, they provided evidence in this mechanism where Us11 exerts a key function in sequestering and stabilizing the duplex region of dsRNA and in blocking downstream recognition by PKR and other pattern recognition receptors (PRRs) that identify dsRNA. However, the ability of VHS to destabilize dsRNA and dsRNA/Us11 complexes raises many questions about the mechanisms involved during viral replication as they specified in their discussion^68^. From our point of view, the finding that VHS controls the activation of PKR raised three main questions: (1) does VHS control PKR through physical interactions? We verified that VHS protein was incapable of interacting physically with PKR (data not shown); (2) is the enzymatic nature of VHS indirectly responsible for PKR control? Many data support this and, in particular, Smiley’s group speculated that VHS might play a more direct role in destroying the dsRNA region by “nibbling” in from one or both ends of the duplex, perhaps with the assistance of an RNA helicase; (3) does VHS recruit other proteins in order to control PKR and facilitate the viral immune escape? To answer to the last question, in the present work, we focused our attention on viral protein kinases UL13 and Us3 of HSV-1, capable of utilizing the phosphorylation system to establish a cellular environment for efficient viral replication. The key question was the extent to which these activities are mechanistically connected. Here, we demonstrate for the first time that both serine/threonine kinase proteins control the accumulation of ph-PKR during HSV-1 replication (Fig. 3). Interestingly, we also discover a potential interplay between VHS and Us3 by detecting a shifted high molecular weight VHS band following transfection with Us3 (Fig. 5). As reviewed by Kato & Kawaguchi 2018, more than 23 phosphorylation events are upregulated by HSV-1 Us3. In particular, UL34, UL31, gB, UL47, vdUTPase, Us8A, Us9 and Us3 itself are physiological substrates of Us3 in infected cells and are directly linked with Us3 functions in infected cells^44^. Based on the above data, we propose that the Us3 kinase activity is responsible for the VHS phosphorylation, and in turn, VHS can exert its RNAse activity on Us3 transcripts and limit the accumulation of Us3 protein. However, the total shutoff of ph-PKR accumulation was detected following pVHS transfection alone as reported also in previous transfection experiments (Figs. 1c and 3c), and seem not to be dependent on VHS phosphorylation events mediated by Us3. Therefore, we highlight the versatility of HSV-1 in the use of several viral proteins to counteract innate immune surveillance. Further investigation will be required to deeply analyse the double regulatory mechanism linking VHS and Us3 proteins and to identify new viral protein–protein interactions during HSV-1 infection.

## Methods

### Cell cultures

Cell lines were originally obtained from the American Type Culture Collection (ATCC). Vero cell lines were propagated in Eagle’s Minimum Essential Medium (EMEM, Lonza, Belgium), supplemented with 6% Fetal Bovine Serum (FBS) (Euroclone), 100 U/ml penicillin and 100 μg/ml streptomycin (Lonza, Belgium). HEp-2 cells and 293T cells were maintained in Dulbecco’s Modified Eagle’s Medium (DMEM Lonza, Belgium) supplemented with 10% FBS, 100 U/ml penicillin and 100 μg/ml streptomycin. SH-SY5Y cells were grown in MEM medium supplemented with 10% FBS, 100 U/ml penicillin and 100 μg/ml streptomycin, 15% of Non-Essential Amino Acid Solution (NEAA) 1X and 20 mM of L-Glutamine. All cell lines were grown at 37 °C in a 5% CO2 incubator.

### Viral infection

HSV-1 strain F is a prototype HSV-1 strain kindly provided by Professor Bernard Roizman (University of Chicago, USA). HSV-1 mutant viruses R2621 (deleted virus for *vhs* gene)^67^, R7041 (deleted virus for *us3* gene)^69^, R7356 (deleted virus for *ul13* gene)^70^ were generous gifts from Professor Bernard Roizman. ΔUL49, deleted virus for *ul49* gene, was generated by Sciortino and colleagues as described elsewhere^35^. Virus stocks were produced and titered in Vero cells. The experimental infections were carried out by exposure of all cell lines to the wild type and/or mutant HSV-1 viruses at the multiplicity of infection (MOI) of 10, according to the experimental design. The adsorption of the virus was carried out for 1 h at 37 °C with gentle shaking. After infection, the supernatant was replaced with fresh culture medium, and then the infected cultures and related controls were incubated at 37 °C, under 5% CO_2_, and collected at the established times of the experimental design.

### Transient transfection assay

293T cells were transfected with the plasmids pVHS, pVHSm, pUS3, pUL13, pUL48 and pUL49, expressing the wild-type VHS, nuclease-defective mutant VHS, U_S_3, U_L_13, U_L_48 and U_L_49 open reading frames (ORFs), respectively, as described elsewhere^36,71–73^. The plasmid pCMS-EGFP, symbolised in the figures as pCMS (Clontech, Palo Alto, CA), was used as a control and added to each sample to ensure that each transfection receives the same amount of total DNA. A total of 5 × 10^5^ cells were seeded in 6-well plates in the presence of DMEM medium supplemented with 10% FBS and 100 U/ml penicillin and 100 μg/ml streptomycin. The next day, the medium was replaced with fresh medium without serum and antibiotics. One μg of each DNA plasmid was incubated with Reagent Plus (Invitrogen) in OptiMEM separately, for 20 min at RT. Lipofectamine (Invitrogen) was then added to the mixture and the two solutions were incubated at RT for 30 min. The DNA-Lipofectamine mixture obtained was slowly added to cultured cells and incubated for 4 h at 37 °C. The medium was replaced with OptiMEM supplemented with 10% FBS. The cells were collected and processed for immunoblot analysis according to experimental design.

### Protein extractions and immunoblot

Immunoblot analysis was carried out to evaluate the accumulation of both viral and cellular proteins. Total proteins were extracted by lysing cells in SDS sample buffer 1X [62.5 mM Tris-HCl (Tris(hydroxymethyl)aminomethane hydrochloride) pH 6.8; 50 mM DTT (dithiothreitol;); 10% glycerol; 2% SDS (sodium dodecyl sulfate); 0.01% Bromophenol Blue], briefly sonicated, boiled for 5 minutes and analysed for protein determination using a QubitTM Protein Assay Kit (InvitrogenTM). An equal amount of protein extracts was subjected to sodium dodecyl sulfate-polyacrylamide gel electrophoresis (SDS-PAGE) and transferred to nitrocellulose membranes (Bio-Rad Life Science Research, Hercules, CA). The membranes were incubated overnight at 4 °C with the appropriate primary antibody and then were probed with secondary antibodies at room temperature for 1 h. Protein bands were visualized by using Immobilon Classico Western HRP substrate (Merk, Millipore) or Super Signal West Pico (Thermo Scientific, Rockford, IL) as chemiluminescent substrates and captured using a ChemiDoc Touch Imaging System (Bio-Rad) where indicated. The quantitative densitometry analysis of each sample was performed by TINA software (version 2.10, Raytest, Straubenhardt, Germany). The intensity of the target protein was divided by the intensity of the GAPDH and graphically represented by GraphPad Prism 6 software (GraphPad Software, San Diego, CA, USA). The experiments were repeated for three times and the numbers are indicated as mean ± SD. Statistical analysis was performed by ANOVA followed by Bonferroni’s Multiple Comparison Test.

### Antibodies and reagents

Polyclonal antibodies against the housekeeping gene GAPDH (sc-32233), GFP (B-2) (sc-9996) and PKR (A12) (sc393038) were purchased from Santa Cruz Biotechnology (Santa Cruz, CA), Anti-PKR (phospho-T446, ab 32036) was purchased from Abcam (Cambridge, England). Antibodies against the viral proteins VHS and U_S_3, were kindly provided by Professor Bernard Roizman. Secondary antibodies anti-rabbit and anti-mouse IgG conjugated to peroxidase were purchased from Merck Millipore. Polyinosinic-Polycytidylic acid [poly (I:C)] was used to induce activation of PKR and was provided by Sigma Aldrich (P1530). 293T cells were transfected as described above and 24 h post transfection, the cells were treated with 10 μg/ml of poly (I: C) diluted in OptiMEM. Samples were then collected at indicated time post transfection to perform immunoblot analysis. Actinomycin D (A9415), Cycloheximide (01810) and Phosphonoacetic acid (P6909) were purchased from Sigma-Aldrich.

### Quantitative Real-Time PCR

HEp-2 and 293T cells were infected and/or transfected according to the experimental design and processed to quantitative Real-Time PCR. Total RNA was extracted using TRIzol® (Life Technologies), according to the manufacturer’s instructions, and DNase-treated before cDNA transcription as follows: 20–50 μg of RNA were incubated at 37 °C for 2 h with 5 μl 10X DNase I Buffer, 2 μl Recombinant RNase-free DNase I (10U) (2270A TaKaRa, Dalian, China) and RNase inhibitor (20U) (N251A Promega). Samples were treated with 2.5 μl EDTA 0.5 M for 2 min at 80 °C and stored in presence of 10 μl sodium acetate 3 M and 250 μl cold ethanol at −80 °C. Next, the samples were centrifuged at 12,000 rpm for 10 min at 4 °C. RNA pellets were then washed with 70% cold ethanol, centrifuged at 12,000 rpm for 5 min at 4 °C, air-dried at RT to remove ethanol and dissolved in a suitable amount of DEPC-treated water. Total RNA was reverse transcribed using ReverTra Ace® qPCR RT Master Mix (FSQ-201 Toyobo). The RT reaction was carried out in Biometra TPersonal PCR thermocycler under the following conditions: 37 °C for 15 min, followed by 50 °C for 5 min and 98 °C for 5 min. The cDNAs were used for quantitative Real-Time PCR. Quantitative Real-Time PCR was carried out on a Cepheid Smart Cycler II System (Cepheid Europe, France) using Maxima SYBR Green (Thermo fisher Scientific). The reaction conditions and the analytic primers to Real-time PCR are listed in Table 1. The cDNA copy numbers were normalized to 18S rRNA. Data represent the mean of 3 biological replicates including a minus-reverse transcriptase control. One-way analysis of variance (ANOVA) and the GraphPad Prism 6 software (GraphPad Software, San Diego, CA, USA) were used to perform statistical analysis and graphical representations, respectively.

**Table 1:** Sequences of Quantitative Real-Time PCR primers.

Gene	Primers	Reaction conditions	
PKR	Fw:5′-CCAGGCAAACAAGGTCCCATC-3′	95 °C 15 sec 60 °C 30 sec 72 °C 20 sec	35 cycles
	Rev: 5′-GCGAGTGTGCTGGTCACTAA-3′		
18S rRNA	Fw: 5′-CTCAACACGGGAAACCTCAC-3′	95 °C 15 sec 60 °C 30 sec 72 °C 35 sec	35 cycles
	Rev: 5′-CGCTCCACCAACTAAGAACG -3′		
VHS	Fw: 5′-ACATAACTGCGGTGCTCTTC-3′	95 °C 15 sec 60 °C 30 sec 72 °C 45 sec	40 cycles
	Rev: 5′-CCGAAATTCTAACCCAACAG-3′		
US3	Fw: 5′-ACTGGCATGGGCTTTACGATC-3′	95 °C 15 sec 60 °C 30 sec 72 °C 45 sec	40 cycles
	Rev:5′-GGAGGACCAGACACGTGACCC-3′		
