# Author Correction: VHS, US3 and UL13 viral tegument proteins are required for Herpes Simplex Virus-Induced modification of protein kinase R

**DOI:** 10.1038/s41598-021-87273-0

**Published:** 2021-03-30

**Authors:** Rosamaria Pennisi, Maria Musarra-Pizzo, Zhixiang Lei, Grace Guoying Zhou, Maria Teresa Sciortino

**Affiliations:** 1 Department of Chemical Biological Pharmaceutical and Environmental Sciences, University of Messina, Viale F. Stagno d’Alcontres, 31, Messina, 98166 Italy; 2 Shenzhen International Institute for Biomedical Research, 140 Jinye Ave. Building A10, Dapeng New District, Shenzhen, Guangdong 518116 China; 3 School of Basic Medical Sciences, Guangzhou Medical University, Guangzhou, Guangdong 511436 China

Correction to: *Scientific Reports* https://doi.org/10.1038/s41598-020-62619-2, published online 27 March 2020

The Acknowledgements section in this Article is incomplete.

“These studies were aided by a grant from University of Messina Research & Mobility 2016 Project (Project Code: RES_AND_MOB_2016_Sciortino) and by a grant from the Shenzhen Science and Innovation Commission Project (Grant number: JCYJ20170411094933148) to Shenzhen International Institute for Biomedical Research. We thank Professor Bernard Roizman for the unequalled discussions in particular about the enrollment of Us3 and UL13 on PKR regulation.”

should read:

“These studies were aided by a grant from University of Messina Research & Mobility 2016 Project (Project Code: RES_AND_MOB_2016_Sciortino) and by a grant from the Shenzhen Science and Innovation Commission Project (Grant number: JCYJ20170411094933148) to Shenzhen International Institute for Biomedical Research. We thank Professor Bernard Roizman for the unequalled discussions in particular about the enrollment of Us3 and UL13 on PKR regulation. “FEAMP 2.56 FISH PATH NET” has contributed to cover the Fee.”

